# EURADOS STRATEGIC RESEARCH AGENDA 2020: VISION FOR THE DOSIMETRY OF IONISING RADIATION

**DOI:** 10.1093/rpd/ncab063

**Published:** 2021-05-15

**Authors:** R M Harrison, E Ainsbury, J Alves, J-F Bottollier-Depois, B Breustedt, M Caresana, I Clairand, E Fantuzzi, P Fattibene, P Gilvin, O Hupe, Ž Knežević, M A Lopez, P Olko, V Olšovcová, H Rabus, W Rühm, M Silari, L Stolarczyk, R Tanner, F Vanhavere, A Vargas, C Woda

**Affiliations:** University of Newcastle, Newcastle, UK; Public Health England, Chilton, Didcot, UK; Instituto Superior Técnico (IST), CTN, Lisboa, Portugal; Institut de Radioprotection et de Sûreté Nucléaire (IRSN), Fontenay-aux-Roses Cedex, France; Karlsruhe Institute of Technology (KIT), Karlsruhe, Germany; Politecnico di Milano, Milano, Italy; Institut de Radioprotection et de Sûreté Nucléaire (IRSN), Fontenay-aux-Roses Cedex, France; ENEA - Radiation Protection Institute, Bologna, Italy; Istituto Superiore di Sanità (ISS), Rome, Italy; Public Health England, Chilton, Didcot, UK; Physikalisch Technische Bundesanstalt (PTB), Braunschweig and Berlin, Germany; Ruđer Bošković Institute (RBI), Zagreb, Croatia; Centro de Investigaciones Energéticas, Medioambientales y Tecnológicas (CIEMAT), Madrid, Spain; Instytut Fizyki Jądrowej Polskiej Akademii Nauk (IFJ PAN), Kraków, Poland; ELI Beamlines, Institute of Physics, Czech Academy of Sciences, Dolní Břežany, Czech Republic; Physikalisch Technische Bundesanstalt (PTB), Braunschweig and Berlin, Germany; Helmholtz Zentrum München, Institute of Radiation Medicine, Neuherberg, Germany; CERN, 1211 Geneva 23, Switzerland; Danish Centre for Particle Therapy, Aarhus, Denmark; Instytut Fizyki Jądrowej Polskiej Akademii Nauk (IFJ PAN), Kraków, Poland; Public Health England, Chilton, Didcot, UK; Belgian Nuclear Research Centre (SCK-CEN), Mol, Belgium; Institute of Energy Technologies, Universitat Politecnica de Catalunya, Barcelona, Spain; Helmholtz Zentrum München, Institute of Radiation Medicine, Neuherberg, Germany

## Abstract

Since 2012, the European Radiation Dosimetry Group (EURADOS) has developed its Strategic Research Agenda (SRA), which contributes to the identification of future research needs in radiation dosimetry in Europe. Continued scientific developments in this field necessitate regular updates and, consequently, this paper summarises the latest revision of the SRA, with input regarding the state of the art and vision for the future contributed by EURADOS Working Groups and through a stakeholder workshop. Five visions define key issues in dosimetry research that are considered important over at least the next decade. They include scientific objectives and developments in (i) updated fundamental dose concepts and quantities, (ii) improved radiation risk estimates deduced from epidemiological cohorts, (iii) efficient dose assessment for radiological emergencies, (iv) integrated personalised dosimetry in medical applications and (v) improved radiation protection of workers and the public. This SRA will be used as a guideline for future activities of EURADOS Working Groups but can also be used as guidance for research in radiation dosimetry by the wider community. It will also be used as input for a general European research roadmap for radiation protection, following similar previous contributions to the European Joint Programme for the Integration of Radiation Protection Research, under the Horizon 2020 programme (CONCERT). The full version of the SRA is available as a EURADOS report (www.eurados.org).

## INTRODUCTION

In 2012, the European Radiation Dosimetry Group (EURADOS) began work on a Strategic Research Agenda (SRA) to identify topics which would influence the development of radiation dosimetry and its applications in a wide range of academic and applied areas for the following two decades, with the fundamental goal of improving radiation protection of radiation workers, patients and the public^(^^1^^,^  ^2^^)^. As dosimetry is a developing subject, periodic revisions to the SRA are needed. The first revision has now been published^(3)^ and can be downloaded from www.eurados.org. This paper summarises its key points.

Since the first SRA, the EURADOS network has continued to expand and now comprises a self-sustainable network of 80 European institutions (Voting Members) such as research centres, university institutes, reference laboratories, dosimetry services and commercial companies, including over 600 active scientists in the field of radiation dosimetry. The aim of the network is to promote European cooperation in research and development in the dosimetry of ionising radiation and its implementation in routine practice—drawing upon European, as well as global, developments—in order to contribute to compatibility within Europe and conformance with international practices.

In pursuing these objectives, EURADOS Working Groups (WGs) continue to cover a wide range of various dosimetric disciplines such as individual monitoring, environmental, internal, retrospective, medical, high-energy and computational dosimetry (www.eurados.org).

EURADOS, through its WGs, has the capacity to develop, test and compare novel dosimetric techniques involving a wide range of participating institutions. This expertise also enables problems arising from new applications of ionising radiation to be addressed and thus to contribute to science-based policy recommendations.

Harmonisation, education and training are also key activities for EURADOS, through the organisation of intercomparisons (e.g. in individual and environmental monitoring, internal dose assessment and computational dosimetry methods)^(4–9)^ and training courses^(10)^.

The current revision of the SRA draws upon the expertise and experience of the WGs^(^^11^^,^  ^12^^)^ and incorporates the results of a stakeholder meeting organised in 2016, with input from over 20 organisations within and outside the dosimetric community^(13)^. The SRA revision has since been discussed at various levels within EURADOS (WGs, Council, Voting Members) by 59 contributors.

EURADOS has been an active participant in the CONCERT project, established in 2014 by the European Commission for the development of a European Joint Programme (EJP) for the Integration of Radiation Protection Research under the Horizon 2020 programme. The objective was to promote the sustainable integration of European and national research programmes in radiation protection. CONCERT operated as a project that brought together research initiatives—and supported SRA development—of several radiation protection research platforms^(14)^: EURADOS (dosimetry), MELODI (Multidisciplinary European Low Dose Initiative), ALLIANCE (European Radioecology Alliance), NERIS (European Platform on Preparedness for Nuclear and Radiological Emergency Response and Recovery), EURAMED (European Alliance for Medical Radiation Protection Research) and SHARE (Social Sciences and Humanities in Ionising Radiation Research). In conjunction with these platforms, EURADOS has contributed to the publication of the Joint Roadmap for Radiation Protection Research (JRM), drawing upon the cross-cutting and underpinning role of dosimetry and including elements from its first SRA. The revised SRA will also be used in future roadmap and prioritisation exercises in a future post-CONCERT structure.

The EURADOS SRA summarised in this paper is made up of five visions, each of which represents a key area of dosimetry development comprising several challenges and, at a more detailed level, associated research lines. The visions, which demonstrate the breadth of dosimetry topics covered by EURADOS, are: updated fundamental dose concepts and quantities (vision 1); improved dosimetry for radiation risk estimates deduced from epidemiological cohorts (vision 2); efficient dose assessment in the case of radiological emergencies (vision 3); integrated personalised dosimetry in medical applications (vision 4) and improved radiation protection of workers and the public (vision 5). In addition, three additional areas are common to, and underpin, the visions, namely computational dosimetry, harmonisation of practice, and education and training. There are significant links between research lines from different challenges, and even between different visions. These cross-cutting links, and many others, are given in the SRA^(3)^.

More information about EURADOS, including a downloadable version of the full SRA, may be found on the EURADOS website (www.eurados.org).

## VISION 1: TOWARDS UPDATED FUNDAMENTAL DOSE CONCEPTS AND QUANTITIES

The biological effectiveness of ionising radiation is believed to be a function of microscopic and nanoscopic energy deposition patterns (particle track structure) involving random interaction events. However, the current radiation protection system is based on protection and operational quantities^(15–18)^ derived from absorbed dose, essentially a ‘point’ quantity, which in practice is averaged over an entire organ or tissue. The overarching objective of this vision is to develop a unified concept of radiation quality which includes the statistical features of track structure. An essential prerequisite for this objective is the identification and quantification of the relevant statistical characteristics of microscopic and nanoscopic spatial and temporal interaction patterns and their correlations with biological damage. This work is, for example, highly significant in the development of hadron radiotherapy and the use of high-Z nanoparticles (see below, and vision 4), but also has a much wider potential influence on dosimetry generally, as described in all five visions. The following three challenges were identified:

### Spatial correlations of radiation interaction events

To improve the understanding of spatial correlations of radiation interaction events, it is necessary to develop a novel, unified concept of radiation quality as a general physical characteristic of the radiation field that would allow the separation of the physical and biological components which contribute to the eventual biological effects of radiation. The aim would be to have a physical ‘dose’ quantity that in the absence of biological variability would give a unique dose–response relationship. This will require several lines of research:

- Develop density scaling relationships for micro- and nano-dosimetry^(^^19^^,^  ^20^^)^ using theoretical and simulation studies as well as the characterisation of existing^(21)^ and emerging nanodosimetric detectors^(22–24)^.
- Identify biologically relevant target sizes from a comprehensive characterisation of track structure; develop track structure imaging techniques^(^^25^^,^  ^26^^)^, complemented by experimental investigations of radiation interactions in condensed phase nanometric objects^(^^22^^,^  ^27^^,^  ^28^^)^.
- Establish uncertainty estimations for measured track structure quantities and develop computational methods for track simulations, including the fundamental challenge of incorporating quantum mechanical descriptions of particle interactions^(^^29^^,^  ^30^^)^.

### Track structure and radiation damage

It has been demonstrated that track structure shows a strong correlation with the induction of early biological effects, particularly the occurrence of DNA single and double strand breaks^(31–33)^. As later biological endpoints also show dependence on radiation quality, correlations between track structure characteristics and the probability of inducing these later effects, such as chromosomal aberrations or cell death, will form the basis of a deeper understanding of radiation damage mechanisms. In general, the prediction of biological effects from track structure characteristics would be a prerequisite for new dosimetric concepts which quantify radiation effects at the level of individual cells or small tissue compartments. Exploration of these correlations suggests several research objectives:

- Study the geometrical correlation of energy deposition and cellular damage using microbeams to target individual cells and small tissue compartments, together with automated assays and metrological methods aimed at improving the detection of radiation-induced biological endpoints^(34–36)^.
- Develop multi-scale characterisation of track structure using nanodosemeters with multiscale measurement capabilities and track structure simulation codes^(^^19^^,^  ^37^^,^  ^38^^)^.
- Identify the most relevant target size for a particular biological endpoint by using correlations between results for a particular endpoint for different radiation qualities, nanodosimetric probability distributions and target sizes.
- Improve the detection of radiation-induced biological endpoints using experimental developments such as transmission electron microscopy for the demonstration of DNA damage^(39)^.
- Investigate high-throughput analysis techniques and transcriptomic profiling of single cells for investigation of intrinsic factors underlying intracellular differences^(^^40^^,^  ^41^^)^.

A further extensive area of research requires a more comprehensive understanding of biochemical reactions and the cellular chemical environment in the production of radio-induced damage (e.g. the role of oxygen in DNA damage and the understanding of the role of various cellular scavenging species). Monte Carlo (MC) techniques need to be developed to predict radio-induced damage in biomolecules. This research is also relevant to the use of high-Z (e.g. gold) nanoparticles (GNP) in radiotherapy^(42–45)^ (see vision 4). The enhanced absorbed doses in the vicinity of multiple GNPs at the cellular and molecular level require verification that is often provided by MC simulations^(46–49)^ which more generally should be extended to include chemical effect modelling for radiobiological simulations. A further challenge is to simulate X-ray fluorescence as a new imaging modality for targeted molecular radiotherapy in parallel with experimental developments^(50)^.

Recent developments in FLASH radiotherapy at high fluence rates^(51)^—where the assumption of the independence of physico-chemical interactions may not be valid—has emphasised the need for the investigation of temporal correlations of interaction events (see also vision 4).

Combining track structure-based nanodosimetry, biologically based mechanistic modelling and epidemiological data should provide insights into the molecular dosimetry required for understanding dose–response relationships at low doses and low dose rates.

### Radiation protection and operational dosimetry quantities

The system of radiation protection employs both protection and operational quantities^(^^16^^,^  ^17^^)^. The protection quantities are intended to estimate detriment, whilst the operational quantities are designed to provide measurable, but conservative, estimates of the protection quantities. The success of this system requires periodic review, revision and verification of these quantities in terms of their applicability, ease of use and relation to detriment. The link between operational and protection quantities would be greatly assisted by making the former relate better to the latter, whilst retaining measurability. However, as the definitions of the quantities evolve, there is a need for the generation of new or revised conversion coefficients as the range of particle types and energies expands, together with the availability of corresponding calibration fields. As concepts change, there is a need to promote research that supports greater understanding of the quantities in the diverse fields in which they are applied. As mentioned in the previous SRA^(1)^, progress in micro- and nano-dosimetry may require revised protection and operational quantities that better reflect radiation damage in the body.

#### VISION 2: TOWARDS IMPROVED DOSIMETRY FOR RADIATION RISK ESTIMATES DEDUCED FROM EPIDEMIOLOGICAL COHORTS

In the context of exposure to ionising radiation, epidemiological studies analyse the rates of observed radiation-induced health and biological effects in a target population and derive the risks of these effects in comparison with background or baseline rates. These investigations involve the collection of exposure and outcome data and provide—in the ideal case—individual dose estimates. The current fundamental quantity for risk estimation is absorbed dose in organs appropriate to the outcome under investigation. Studies are performed on cohorts comprising humans exposed, amongst others, to emergency, medical and occupational exposure situations. The dosimetric challenges in these situations are described in visions 3, 4 and 5, respectively. Despite the diversity of exposure conditions, they contain considerable overlapping approaches and methodologies for absorbed dose evaluation. Some of the most important objectives are:

- Assess doses independently for each quality (e.g. in mixed photon and neutron fields).
- Estimate doses which had originally not been recorded (e.g. out-of-field doses in radiotherapy).
- Identify plausible sources or pathways to exposure and exclusion of those less significant (e.g. cross-fire from non-target organs).
- Use historical dose records, including their validation and retrospective re-evaluation.
- Collect auxiliary data (e.g. equipment types, measurement protocols, workloads).
- Apply dose estimation and reconstruction methods, including data reliability, recalibration and uncertainty estimation.

Dosimetric support to epidemiological studies has three major objectives: first, to provide dose estimates with minimum bias for all cohort members and exposure sources; second, to provide uncertainty estimates for all doses; and third, to validate dose estimates by independent benchmarking exercises.

In spite of considerable work in these areas, there are several improvements and developments which need to be made and these are described in the following three challenges:

### Improvements to dosimetric data

Most epidemiological studies on exposed cohorts (e.g. A-bomb survivors, Chernobyl and Techa River populations, medically irradiated patients) are retrospective and doses need to be evaluated *a posteriori*, often with sub-optimal data. Also, new dosimetric challenges arise when cohorts are pooled to increase statistical power, since this requires dose estimates from diverse global origins to be harmonised and risk estimates adjusted for dose uncertainties. Several lines of investigation are indicated as follows:

- In a particular retrospective study, doses to organs and tissues may be required which will not have been included in the original dose monitoring or assessment procedures. For example, in medical exposures, doses to the organs and tissues of interest may differ significantly from values estimated and recorded in the course of routine application of the respective medical procedures. Non-target absorbed doses in radiotherapy are also difficult to determine retrospectively from recorded doses. In personal monitoring, whole body dose is inadequate for the estimation of individual organ doses. To address these problems, dosimetric methodologies (e.g. MC transport calculations, biokinetic models, data aggregation), the recovery and use of available initial data and the reconstruction of missing information (e.g. workloads) should be combined and further elaborated.
- In many cases, the quality of the initial data for dose reconstruction requires improvement. For example, personal monitoring data may be improved by re-evaluation of historical records and ‘recalibration’ of the historical dosemeters using MC simulations. Further problems are associated with handling data from very large cohorts (e.g. tens of thousands), estimating doses over time frames within which exposure conditions may have changed, lack of key data, and dose measurements which may be below detection or recording levels. Software for dose estimation is currently specific to particular cohort studies and more flexible and adaptable software is needed, particularly for use with very large data sets.
- The development of more realistic biokinetic and dosimetric models would be valuable. These include age- and gender-specific biokinetic models, new mesh-type computational phantoms, hybrid phantoms combining both voxel-based and simplified equation-based modelling approaches and the generation of hybrid computational phantom libraries for internal radiation dosimetry (see also vision 3 and the section on Computational Dosimetry).

### Uncertainty estimation and dose validation

The estimation of dose uncertainties has previously used simplified analytical models. However, the recent development of stochastic risk models requires well-established dose uncertainty distributions, which can be used as input to MC risk calculation algorithms. Generally, improvements in uncertainty assessments are likely to enhance the confidence in derived dose–response functions and increase the statistical significance of the results obtained.

Validation of dose estimates by comparison with independent measurements will continue to be important but requires further development, building upon instrumental developments such as electron paramagnetic resonance (EPR) on tooth enamel and fluorescent *in situ* hybridisation (FISH) on circulating lymphocytes. The reduction of dose uncertainties in epidemiological studies is challenging. Systematic quantification and harmonisation of data uncertainties is first required, followed by an analysis of their influence on the results. Statistical methods should continue to be developed to account for the complex nature of errors in risk analyses, following initial successful applications in several studies.

### Future epidemiological studies

Finally, it is important to anticipate future epidemiological studies. Although many such studies are retrospective in nature, the prerequisites for future studies may differ, for example in the investigation of health effects caused by novel technologies (e.g. ion beam radiotherapy). Detailed descriptions of doses, their estimation and the technologies used in each exposure scenario are needed to anticipate the data required, together with multidisciplinary development of expanded data registries. The emergence of molecular epidemiology requires new standardised dosimetry and scoring methods to support harmonisation of dosimetric practices.

## VISION 3: TOWARDS EFFICIENT DOSE ASSESSMENT IN RADIOLOGICAL EMERGENCIES

Radiological emergencies are a major challenge in modern society. They include three distinct types of incidents:

- Those that have an impact on large geographical areas and lead to the exposure of large groups of the general population such as at Chernobyl and Fukushima.
- Accidents that involve industrial or medical radiation sources, usually involving a relatively small number of victims.
- Terrorist attacks which may involve radioactive materials, either in the form of radiological dispersal devices (‘dirty bombs’), containing radioactive materials in addition to conventional explosives, or radiological exposure devices (sealed hidden sources).

Each of these incident types is associated with specific problems in determining radiation doses, identifying individuals who are at the highest risk and deciding the best method to be applied for evacuation, medical treatment and remediation. The dosimetric protocols and techniques employed will depend, in particular, on the number of victims and the severity of the exposure. As a first stage, triage is important, followed by more precise dose investigations of identified exposed individuals.

In most incidents, a quick, efficient and reliable estimate of doses to affected individuals or groups of individuals is a prerequisite for further decision-making by responsible authorities. Dose assessment is complicated by the fact that a number of concurrent exposure scenarios might be of concern, e.g. internal exposures from incorporated radionuclides together with external exposures from various sources. Real-time (environmental) monitoring (in the case of nuclear power plant accidents) or dose-rate measurements by various approaches (manually, stationary, car-borne, air-borne) is usually the first step in assessing doses to population groups and identifying critically exposed sub-groups. Due to the availability of affordable dose-rate metres for the public, citizen networks are becoming an increasingly relevant aspect in cases of public exposure.

Improvements are needed in the application of methods for individual dose measurement, enabling decision makers to reassure the ‘worried-well’ rapidly, to identify individuals with a high risk of developing radiation-induced injuries, and to initiate the most urgent actions, including methods of reducing doses after internal contamination. Incidents which have an impact on large geographical areas and populations require the handling and processing of a large number of samples in a short time, whereas for those affecting a relatively small number of victims (e.g. in industry or medicine), an accurate organ-specific dose assessment should be available.

Dose assessment is also relevant for the long term and recovery phases, where it should support epidemiological studies to evaluate the possible health impact on affected populations (see vision 2), address the needs of individuals and society and support the establishment of health surveillance programmes.

Four main challenges have been identified:

### Quantification of doses from internal emitters

New equipment and methods for *in vivo* and *in vitro* monitoring of radionuclides incorporated in the body are required^(52)^. These include the development of validated MC methods using age-scaled computational phantoms (voxel or mesh phantoms) for the calibration of body counters, assessment of internal doses, particularly for children and the improvement of emergency *in vitro* radiobioassay. Development of standard, validated, protocols and optimisation of equipment use and dose assessment is also required, including citizen involvement where appropriate. In parallel with this, the development of new biokinetic modelling for human and non-human biota is needed, including DTPA therapy modelling in the case of administration of this decorporation agent in persons exposed to actinides. Linking also with vision 2, epidemiological studies require the development of new methods for dose reconstruction through improved standard methodology and computational tools for the calculation and use of internal doses in cases involving internal (and mixed internal plus external) exposures. The application of biodosimetry methods in cases of accidental intake of radionuclides should be investigated, as well as the improvement in monitoring and dose assessment in cases of wound contamination.

### Individual doses from external exposure

A second complementary challenge is to improve the quantification of individual doses from external exposure in emergencies^(53)^. Standardisation of existing consolidated markers of physical and biological dose assessment should be continued through inter-laboratory comparisons, field tests and by more accurate investigation and quantification of uncertainties^(^^54^^,^  ^55^^)^.

Further exploration is needed of the properties of materials which comprise personal items used as fortuitous physical dosemeters (e.g. mobile phones) with EPR, thermoluminescence (TL) and optically stimulated luminescence (OSL), together with new biomarkers including the gamma-H2AX assay and gene expression^(56)^. The emphasis should be on increasing sensitivity, decreasing uncertainties, improving capacity and decreasing the processing time.

The identification of new markers of radiation exposure is important, especially for quick identification of individuals in potential danger of short-term deterministic health effects (tissue reactions) in the early phases of a radiation incident. Here, the emphasis is on methods for rapid dose measurement with high throughput. The establishment and maintenance of an international network of experienced laboratories in retrospective dosimetry becomes essential.

### Improvement of environmental monitoring

A third challenge is to improve environmental monitoring in the event of an accident, including the use of mobile networks, collaboration with citizens and harmonisation of methods. New detection systems for environmental monitoring in the event of a radiological accident have been developed during the last few years, such as the use of drones and citizens’ networks. In addition, novel environmental spectrometric detectors have been characterised and tested in environmental conditions^(^^57^^,^  ^58^^)^. These developments should be expanded to include alpha emitter detectors, source localisation and imaging systems. This approach will generate a very large amount of extra data for which quality control (QC) systems and harmonisation across several extended data networks will be required. Artificial intelligence techniques are expected to play a significant role.

### Challenges and opportunities of citizen engagement in dosimetry

Finally, opportunities for citizen engagement in dosimetry are emerging^(59)^. These will require the development and assessment of accessible, user-friendly, accurate and reliable tools with clear background information and instructions, to enable citizens to perform their own radiation measurements and enable comparison with other measurements. Involving citizens in ongoing research together with dosimetry experts, social sciences and emergency response experts and authorities in multidisciplinary projects, will aim to maximise the impact of citizen science in dosimetry and to increase the understanding and trust between the different stakeholders in radiation protection and emergency response.

## VISION 4: TOWARDS INTEGRATED PERSONALISED DOSIMETRY IN MEDICAL APPLICATIONS

Diagnostic and therapeutic medical exposures are responsible for ~20% of the average annual dose to the world population from all sources. In diagnostic radiology, including nuclear medicine, ~670 million X-ray examinations per annum occur within the EU with individual patient effective doses ranging from <10 μSv for dental exposures to 15 mSv for CT examinations, the frequency of the latter increasing steadily over recent years. Thus, the collective population dose is considerable and programmes for optimising these exposures are conducted in many countries.

In developed countries, approximately half of all cancer treatments will involve radiotherapy. Healthy tissues outside the target volume may receive doses which vary over several orders of magnitude and considerable work has been undertaken in recent years to quantify and optimise these unwanted doses. The development of dosimetry techniques and the measurement of doses, particularly to radiation-sensitive organs, is an important prerequisite to ensure that patients receive optimum doses, to provide robust dosimetric data for epidemiological studies in diagnosis and therapy (see vision 2), and more generally to advance the understanding of radiation effects on humans.

The principal aim of medical irradiation is to enable an overall benefit to the patient for minimum risk. Thus, dosimetry plays a fundamental role in the justification of radiation exposures; in diagnosis, this requires obtaining the necessary clinical information for the lowest dose, and in therapy, delivering the prescribed dose for acceptable doses to non- treatment organs and tissues. In both diagnosis and therapy, new clinical technologies will lead to new dosimetric challenges. The following three challenges were identified:

### Patient and ambient dosimetry in radiotherapy

In radiotherapy, all stages of the treatment chain involve dosimetry, from radiation generation in the treatment machine and associated QC, to the deposition of dose within the patient, both within the target volume for therapeutic effect, but also within outlying critical radiosensitive organs and tissues (an unwanted component). Dosimetry in the treatment chain needs to be harmonised between treatment centres and this requires the development of dosimetry intercomparisons and audit programmes, particularly for emerging treatments such as proton and ion beam radiotherapy. There is also a need to develop and support the links between nano-, micro-, and macro-dosimetry, and radiobiology and to develop a common conceptual framework (see vision 1). Ultimately, the late effects of radiotherapy are tested by epidemiological studies. For these, the total dose to the organs and tissues from all sources of radiation (therapeutic and diagnostic) is ideally required, but in practice, such comprehensive data may not be available and strategies for risk estimation may currently be inhibited by incomplete dosimetry data (see also vision 2).

More specifically, the development of new and modified dose-delivery systems in radiotherapy requires commensurate developments in associated dosimetry. Amongst the many developments, the introduction of proton and ion-beam radiotherapy is prominent within Europe. Several aspects require further investigation:

- Improve the dosimetry of particle beams (e.g. proton or carbon ions) by the spatial determination of primary particles and their LET for realistic beam intensities. This is important because LET is one of several factors influencing RBE, which has been used in proton therapy planning to allow for variations in the biological effectiveness of protons relative to photons (see also vision 1).
- Develop dosimetry at high fluence rates where interactions may not be temporally independent (see vision 1), particularly active dosemeters suitable for use in the clinical environment (e.g. for short ultra-high dose irradiation (FLASH), which has been shown to be a promising modality for cancer treatment which reduces damage to normal tissue).
- Develop dedicated phantoms to enable acceptable treatment plan verification (e.g. for spot scanning proton arc therapy which offers low entrance doses and high target dose conformity).
- Address fundamental problems in neutron dosimetry in mixed neutron, proton and photon fields (see also vision 5).
- Develop dosimetric QC techniques (as part of a quality assurance (QA) programme) for checks at all stages of the radiotherapy dosimetry chain for on-line validation of dosimetry during treatment. This includes stages involving beam production, spatial and temporal dose delivery, *in vivo* dosimetry and real-time organ dose estimates, small field and edge-of-field dosimetry and dosimetry in magnetic fields.
- Design dosimetry techniques to support the development and validation of models of induced cancer and non-cancer (e.g. cardiovascular) radiation effects and to provide experimental dosimetric validation of treatment planning system dose algorithms.
- Develop dosimetry techniques within a metrological framework for the measurement and estimation of the total dose to the target and critical organs from all radiation sources (therapeutic and diagnostic) to patients receiving radiotherapy. These data are required for more accurate input to epidemiological studies (vision 2) and benchmarking mathematical models for organ dose estimation.

### Patient dosimetry in nuclear medicine

The dosimetry of radiopharmaceuticals is a multi-step process. The key steps in obtaining accurate dose estimates are: (i) measurement of the activity to be administered, (ii) quantitative assessment of the spatial and temporal activity distributions in cells or tissues and (iii) calculation of the deposited energy from these activity distributions.

In nuclear medicine, 90% of the procedures are diagnostic. Currently, the estimated absorbed dose to organs and tissues in patients undergoing diagnostic nuclear medicine examinations is derived from calculations based on reference models of the human body and the biokinetic behaviour of the radiopharmaceutical. Improvements in patient dosimetry in this field will require advances in patient-specific quantitative imaging and individualised biokinetic modelling.

Molecular radiotherapy (MRT)—the use of radiopharmaceuticals for treating cancer (and some non-cancer conditions)—is increasing and the number of MRT clinical trials is expected to increase in the future. In general, improvements in the accuracy of absorbed dose to critical tissues would provide a more effective targeted use of MRT. As an example, the characteristics that make high-LET (e.g., alpha and Auger effect) radiation attractive for targeted therapy also raise several challenges for dosimetry. Knowledge of the non-uniform distribution of the radionuclides (e.g. due to heterogeneous target expression amongst cancer cells and the diversity of structures in the actual tissues of interest) in combination with the short path length and high LET is important for accurate dosimetry.

To address these issues, several objectives may be identified:

- Develop cellular dosimetry models together with radiobiological experiments to assess the intracellular activity distribution and relevant biological endpoints. Currently, accurate knowledge of the dose given to cellular targets of interest is lacking and leads to only approximate correlations with biological end points.
- Develop pre-clinical computational dosimetry to improve the accuracy of dose estimates in pre-clinical models at the organ and sub-organ level. The current assumptions of uniform distributions of radioactivity across entire organs may misrepresent local regional doses to specific organ substructures.
- Develop new computational models which can distinguish between different substructures within organs or tissues of interest (e.g. using imaging modalities such as microCT and microMR).
- Study dose-effect relationships for internal emitters to complement those for external beam radiotherapy. Investigate the justification for applying quantities such as biologically effective dose (BED) and equivalent uniform dose (EUD) to radionuclide therapy.
- Develop quantitative multimodality pre-clinical and clinical imaging for gamma, beta and alpha-emitting radiopharmaceuticals to gain more detailed knowledge of the corresponding patient-specific biokinetics of radiopharmaceuticals for dose assessments in clinical MRT.
- Update biokinetic data and *S*-values for the calculation and optimisation of patient doses in diagnostic nuclear medicine, by implementing more realistic human voxel phantoms.

### Patient dosimetry in CT and interventional radiology

The ubiquitous use of diagnostic X-rays in medicine has led to the requirement for patient dose estimation over a wide range of examinations and doses. Over 660 million examinations are performed annually in Europe with doses varying over four orders of magnitude. In this SRA, we concentrate on two aspects, interventional radiology (IR)—including interventional cardiology (IC)—and computed tomography (CT), both of which can be complex and lead to relatively high patient doses. In IR and IC, doses could potentially be sufficiently high to cause tissue effects, especially if the use of radiation is non-optimised. Currently, measured dose quantities (e.g. entrance skin dose (ESD), dose area product (DAP)) are often insufficient for accurate estimation of organ and sub-organ doses using generic conversion coefficients. An improved system of organ dose estimation is needed based on actual patient anatomy. Computational methods must therefore be developed, based, for example, on predetermined phantom libraries and/or actual patient anatomy. These would enable improved use of diagnostic reference levels (DRLs), achievable dose levels (ADLs) and skin dose alert (trigger) levels for the optimisation of patient doses, improved accuracy of skin and other organ doses, and population dose estimation. The following specific lines of investigation have been identified:

#### Skin and organ doses in IR and IC

- Develop skin dose mapping software to determine real-time skin dose distributions and alert levels, to prevent skin injuries in IR. These calculations need to be validated by measurements.
- Develop reliable real-time skin dose assessment methods together with high spatial resolution modelling of the skin.
- Harmonise DRLs and trigger levels for maximum acceptable skin doses (in both IR and IC). In IC, develop methods for obtaining relevant organ doses (from all imaging modalities used), following reports of radiation-induced effects on the cardiovascular system.

#### Patient-specific dose estimates in CT imaging

CT examinations comprise ~55% of all X-ray examinations in Europe and often lead to some of the highest patient doses, sometimes generating controversy. In future, a transition from scanner beam dosimetry to patient-specific dose estimates is anticipated. The following objectives have been identified:

- Develop real-time methods to match patient image and dose data to those in libraries of realistic computational phantoms to enable organ dose (and dose uncertainty) calculations, whilst retaining the flexibility to accommodate future developments in detector technology.
- Develop new approaches to image quality assessment, a key component of dose optimisation. Optimise imaging protocols for individual patients and assess the reliability and relevance of the data in a multidisciplinary environment, taking into account individual radiation sensitivity and the development of personalised dosimetry.

Although future developments in CT and IR have been emphasised here, the suggested innovations, based on the trend to personalised medicine coupled with extensive computational dosimetry support, are in principle applicable to a greatly extended range of diagnostic radiology examinations.

## VISION 5: TOWARDS IMPROVED RADIATION PROTECTION OF WORKERS AND THE PUBLIC

Methods of radiation protection of workers and the public and subsequent operational implementation has been the subject of active research over recent years. However, further development and a better understanding of some aspects are still needed, for example in the biokinetics of internal contamination pathways, on-line personal dosimetry, neutron dosimetry, radon exposure and space dosimetry. The following five challenges were identified:

### Biokinetic and dosimetric models for internal emitters

Internal doses may occur when unsealed radionuclides are handled by workers in a variety of workplaces (e.g. the nuclear industry, biomedical research and hospitals). The public can also receive internal doses if they are exposed to radionuclides such as radon and its progeny, or from releases of other radionuclides into the environment. In these cases, personal risk is assessed by measurements of incorporated radionuclides supported by applications of biokinetic and dosimetric models which describe the spatial and temporal distribution of radionuclides in the body and their excretion, and the absorption of energy emitted following their decay (see also vision 3). The quantification of internal exposures is supported by *in vivo* monitoring (measurements of radiation emitted from the body by incorporated radionuclides), *in vitro* monitoring (measurements of radionuclides in excreta) and workplace monitoring (measurements of radionuclides in air samples).

Improvements are required in the following areas:

- Improve the measurement of incorporated radionuclides by applying 3D printing technology to enable more realistic calibration phantoms to be produced, supported by parallel developments in numerical calibration techniques. Additionally, *in situ* monitoring should be developed for internal dose assessment where frequent monitoring of workers exposed to short-lived radionuclides is needed.
- Develop a better understanding of the relative contributions to the uncertainty in internal dose estimations. Advances in microscopic biokinetics and micro-dosimetry are required to describe the dose distribution at the individual organ level, where dose homogeneity cannot be assumed.
- For epidemiological studies where life-long personalised dose assessments for cohorts of workers are required, develop cohort-specific software for very large and diverse input data sets, together with associated dose uncertainty estimates (see also vision 2).

### Real-time external personal dosimetry for workers

Over one million workers are exposed to ionising radiation in Europe. The diverse range of exposure situations includes the type and energy of the radiation and the location, size and radiosensitivity of the body parts which may be exposed. For workers exposed to external sources of radiation, a coherent monitoring programme requires the development of reliable, accurate and real-time personal dosimetry from which limiting dose quantities may be derived for the optimum application of protection principles. Several research objectives may be identified:

- Develop on-line (real time) personal dosimetry systems, exploiting new dosemeters associated with connected technologies (i.e. those allowing ‘smart’ connections with other internet-accessible devices). Make comparisons with current systems in terms of accepted performance standards as well as novel features such as the impact of immediate dosimetric feedback. In the longer term, the development of MC-based simulations of exposure situations, together with tracking devices, may complement or even replace current physical methods.
- Test and calibrate active personal dosemeters (APDs), e.g. in medical fluoroscopy-guided procedures, under realistic conditions. Propose algorithms for double dosimetry to assess both the effective dose and the eye lens equivalent dose, including assessment of the potential impact of the new operational quantities for external radiation and ICRP 103 tissue weighting factors^(18)^. More generally, the question of whether APDs can be used as legal dose recorders of whole-body individual dosimetry should also still be addressed.
- Develop and assess eye lens dosemeters and their operational use (including reference doses) and improve and test protection measures such as lead glasses. The reduction in the dose limit for the lens of the eye makes the eye dose potentially the dominant limiting quantity.
- Improve dosemeter characteristics (e.g. low-energy performance) and develop active extremity dosemeters. Extremity exposure in nuclear medicine is a developing field, especially with the use of radionuclides such as ^177^Lu and^68^Ga, where mixed field dosimetry including positrons requires investigation. Whole-body dosemeter positioning, particularly for heterogeneous fields and where partial shielding is employed, is also a topic requiring further investigation. Reports of specific health effects arising from brain irradiation, particularly in medical radiology, require dosimetry for this organ to be re-evaluated and improved.

### Neutron dosimetry

Neutrons are an increasingly important component of workplace radiation fields where accelerators are used for research and medical applications. The neutron spectrum around accelerators is similar to that found at commercial aircraft altitudes, due to the interaction of cosmic rays in the atmosphere. The high-energy component poses a calibration problem because of the very limited availability of reference neutron spectra above 20 MeV^(60)^. The following research objectives may be identified:

- Investigate dosemeter response in workplace fields^(61)^ for medical applications, especially proton and ion-beam radiotherapy, where problems in neutron dosimetry arise in pulsed neutron fields and mixed radiation fields which include neutrons (see vision 4). Some new techniques of particle acceleration based on ultrashort (femtosecond) laser bursts are capable of exploiting the FLASH effect (see vision 1)^(62)^.
- Carry out intercomparisons of radiation exposure codes and their experimental verification^(63)^, especially in the presence of solar storms^(64)^. Neutrons are also the dominant component of the radiation field in commercial aviation^(65)^ and are responsible for doses to air crew^(66)^ which are currently assessed using *ad hoc* computer codes^(67)^.
- Develop improvements to, or replacements for, contemporary neutron dosemeters such as albedo luminescence and etched-track detectors which have inferior performance compared with photon dosemeters for personal monitoring^(^^68^^,^  ^69^^)^.
- Develop new personal dosimetry methods for high-energy fields, e.g. LET spectrometry with PADC^(70)^. Such improvements may also be extended to aircrew^(64)^. Some necessary general improvements to neutron personal dosemeters (and neutron survey dose rate instruments) include a more uniform energy response, directional independence, real-time readout and photon discrimination. Many of these would be enhanced by MC modelling of the appropriate neutron fields.
- Advance the modelling of neutron fields, in conjunction with anthropomorphic phantoms and the development of instrumentation, to allow a direct estimation of neutron effective dose to be made in the workplace. Such developments should include improved performance across a wide range of neutron energies, a detailed knowledge of neutron cross sections at high energies and reduced directional dependence. All the above objectives would benefit from increased availability of high energy quasi-monochromatic reference neutron fields, which are currently lacking in Europe.

### Environmental monitoring

When applied to environmental monitoring of radon, the implementation of directives and guidance on the occupational intake of radionuclides reflects a shift in emphasis from radon activity measurements towards dose estimation. This will require further steps as follows:

- Improve dose estimation for the inhalation of radon, thoron and airborne progeny, taking into account atmospheric and personal inhalation parameters.
- Include the environmental monitoring of radon in environmental climate networks, for example, the use of ^222^Rn as a tracer for the study of greenhouse gas dynamics^(71)^. This would also enable the generation of radon maps for radiological protection purposes. The emergence of novel environmental technologies such as citizens’ networks and drone-mounted detectors requires appropriate characterisation, calibration and validation.
- Advance the metrology of radon by developing environmental detectors for more precise estimation of dose due to radon inhalation in workplaces and dwellings, improving reference sources and calibration for low concentrations, and developing methods of identifying radon priority areas using spectrometric detectors in surveillance networks and aerial measurements.

### Dosimetry in space

Radiation exposure during human spaceflight incurs generally higher doses to astronauts compared with those on Earth. Radiation in space includes a complex mixture of particles and energies and comprises two major components, galactic cosmic rays comprising mainly protons, but also heavier nuclei with energies from a few eV to ~10^20^ eV, and solar cosmic rays, also mainly protons with energies from ~ 10 MeV to 10^4^ MeV. The radiation field in space (energy and fluence spectra) varies with several factors, including the solar cycle, solar activity, planetary atmospheres and surfaces, planetary magnetic fields and spacecraft construction materials. The challenge is to advance the dose measurement capability in space by providing accurate information (spectra, dose rates and microdosimetric quantities) in each exposure situation, taking into account the different radiobiological quality of cosmic rays in space compared with high aircraft altitudes. The following research lines are suggested:

- Develop instrumentation, including radiation spectrometers able to measure, separately, directly and indirectly, ionising particles in a mixed high-energy field. Calibrate detectors in reference fields and cross calibrate instrumentation *in situ*.
- For astronaut safety, improve the prediction of a solar proton event (time of occurrence, fluence spectra, duration) by the development of models (incorporating more comprehensive interaction cross sections) to describe the radiation environment and its interaction with spacecraft.

In addition to the five visions and their respective challenges and research lines, there are three additional areas important to all visions: computational dosimetry, harmonisation and practice, and education and training.

## COMPUTATIONAL DOSIMETRY

Computational methods play an important role in many of the areas of research and development described in the five visions. They are mainly, but not exclusively, based on MC simulations and include dose-distribution simulations, neutron spectral unfolding methods, representations of the human body at macroscopic and microscopic scales, the calculation of operational quantities in radiation protection, studies of energy deposition patterns and the design, optimisation and analysis of experiments. The following are some fields with growing needs for computational dosimetry:

- Operational dose quantities, recently revised by ICRU^(72)^, rely exclusively on anthropomorphic phantoms for their definitions. Computational dosimetry developments are required for the evaluation of the new conversion coefficients and the study of modifications to the design of current dosemeters.
- In the longer term, individual monitoring frameworks should use dose quantities based on, for example, age-dependent phantoms and more detailed models of the body and its organs (see also vision 5).
- Monitoring individuals’ doses in real-time by computational methods is now a possibility and in personalised medicine, patient-specific dosimetry will depend on computational techniques, e.g. in computed tomography, interventional procedures and radiotherapy (see vision 4).
- Individualised computational phantoms are also needed in internal dosimetry where neural networks may be used for the construction of such phantoms and real-time dose estimations (see vision 3).

Recent phantom developments, such as mesh or non-uniform rational B-spline phantoms, permit adaptation to individual personal anatomy. Currently, only a few MC codes are capable of directly incorporating these types of phantoms without the need for voxelisation. Therefore, extension to more codes is required, together with the development of new variance reduction techniques. In the framework of real-time computational dosimetry, the implementation of methods to perform ‘dynamic’ calculations in the MC codes are needed, e.g. to model movement sequences of anthropomorphic phantoms. This is not feasible with voxelised phantoms.

Fundamentally, there is a need for new basic interaction cross sectional data in several fields, including individual monitoring and medicine (new particle types, new radionuclides, high Z nano-particles) and micro- and nano-dosimetry (cross sections for detector materials).

Uncertainty assessment in computational dosimetry is of continuing importance, including code intercomparisons and analysis of the sensitivity of the output to code parameters. In all the above computational applications, experimental benchmarking is indispensable.

## HARMONISATION AND PRACTICE

The goal of harmonisation of dosimetric procedures in Europe is central to the overall EURADOS mission and for accomplishing the strategic objectives in each of the five visions described above. In this context, harmonisation means the achievement of uniform and consistent reliability and overall accuracy of dosimetry even though techniques and other details may differ. For example, dosimetry carried out in different countries may use different techniques and be subject to different national requirements, but the results should be equally reliable and equally accurate. Work on harmonisation cuts across the various visions in several ways: the promotion of intercomparisons enables participants to analyse and improve performance and allows the validation of methods. Surveys enable the radiation protection community to understand current contexts and the publication of agreed recommendations, guidance and interpretations, enables professionals to improve practice.

Specifically, for several visions described above, further harmonisation actions are needed. These include:

- For vision 3: Intercomparisons for (i) retrospective dosimetry methods, (ii) *in vivo* monitoring and MC simulations for accidental intakes of radionuclides and (iii) *in vitro* emergency bioassays.
- For vision 4: (i) harmonisation of methods for nano-, micro-, and macro-dosimetry and their integration with radiobiology, (ii) intercomparisons of dosimetry methods in high dose rate and pulsed medical fields, (iii) harmonisation of dose calculations, measurements, intercomparison protocols and audits in radiotherapy especially for new techniques, (iv) multi-institutional harmonisation of medical imaging codes of practice and (v) intercomparisons for dose calibrators in nuclear medicine.
- For vision 5, intercomparisons for (i) individual occupational monitoring especially for neutron and extremity/eye lens dosimetry (ii) internal dosimetry (*in vivo* and *in vitro* monitoring and dose assessment) and environmental monitoring.

Additionally, for computational dosimetry, further intercomparison exercises are needed for MC simulations and neutron unfolding procedures, amongst others.

## EDUCATION AND TRAINING

Education and training (E&T) have always been key components in EURADOS activities. By means of training courses, dosimetry intercomparisons and networking activities, EURADOS promotes the maintenance and sustainability of radiation protection expertise, in which activities are often carried out by small numbers of highly specialised staff.

The general objectives of EURADOS E&T actions remain the same as reported in the previous SRA. These are:

- To maintain competence in the field of dosimetry, in Europe, especially with new dosimetric techniques.
- To improve the dissemination of information and education of the general public, especially for key groups (e.g. physicians, teachers, journalists, representatives of local authorities), to promote a better understanding of ionising radiation terminology and the development of preparedness programmes for accidents and emergencies.
- To coordinate E&T efforts and initiatives with other platforms and organisations and to contribute to international E&T actions in radiation protection and dosimetry. Previous EURADOS E&T actions have been reported by Alves *et al*.^(10)^.

EURADOS initiatives include:

- The EURADOS Grant, supporting research projects developed at laboratories within the EURADOS network.
- The EURADOS Award for excellence in work developed within a EURADOS WG.
- The Learning Network was established in 2017, as a new tool for E&T, giving participants opportunities to discuss a range of relevant topics on Individual Monitoring at the Annual Meeting.
- A Winter School at the EURADOS Annual Meeting.
- Regular training courses on radiation dosimetry and monitoring.
- Webinars to disseminate Working Group research results.

Coordination with other European platforms for E&T has been realised within the CONCERT project, thus avoiding duplication with other international initiatives, promoting collaborations, and identifying the contribution of EURADOS internationally. Examples include sessions at the European Radiation Protection Week, ICRP meetings, the European Education and Training in Radiation Protection workshop (2019), training courses within the Advanced Networking for Nuclear Education and Training and Transfer of Expertise network and the European Metrology Network.

Additionally, EURADOS supports the dissemination of knowledge in radiation dosimetry by promoting and endorsing conferences such as the individual monitoring (IM) series, the neutron and ion dosimetry symposia (NEUDOS) and, more recently, by facilitating the participation of young scientists in international conferences (EURADOS Young Scientist Conference Support).

With rapid increases in the applications of computing, machine learning and MC techniques, numerous computational dosimetry codes are in use or under development. The organisation of code intercomparison exercises and training courses is an important task for the future, as is indicated in the Computational Dosimetry section.

## SUMMARY

The development of the EURADOS SRA has demonstrated that the understanding, application and development of radiation dosimetry are vital foundations for the entire range of global dosimetry applications. The scope of the SRA is broad, from basic radiation interaction science to practical applications with profound health and societal consequences. Although the diversity of applied dosimetry is clear, some fundamental developments may help to underpin applications in several different fields. For example, advances in internal dosimetry are applicable to nuclear medicine, radiological emergencies and personal monitoring. Similarly, external dosimetry developments may be applied to, amongst others, medical radiology and personal monitoring. The development of computational dosimetry and simulation is proving to be a major boost to all areas of dosimetry and facilitates many theoretical studies and their experimental verification.

To achieve the visions outlined in this paper requires collaboration on a multinational scale, with harmonisation of best practices underpinned by education and training. EURADOS contributes actively to this work and seeks to collaborate as widely as possible to achieve the goals outlined in this paper.
